# Quantitative proteomic analysis of amastigotes from *Leishmania (L*.*) amazonensis* LV79 and PH8 strains reveals molecular traits associated with the virulence phenotype

**DOI:** 10.1371/journal.pntd.0006090

**Published:** 2017-11-27

**Authors:** Eloiza de Rezende, Rebeca Kawahara, Mauricio S. Peña, Giuseppe Palmisano, Beatriz S. Stolf

**Affiliations:** Department of Parasitology, Institute of Biomedical Sciences, University of São Paulo, São Paulo, Brazil; Institut national de la recherche scientifique, CANADA

## Abstract

**Background:**

Leishmaniasis is an antropozoonosis caused by *Leishmania* parasites that affects around 12 million people in 98 different countries. The disease has different clinical forms, which depend mainly on the parasite genetics and on the immunologic status of the host. The promastigote form of the parasite is transmitted by an infected female phlebotomine sand fly, is internalized by phagocytic cells, mainly macrophages, and converts into amastigotes which replicate inside these cells. Macrophages are important cells of the immune system, capable of efficiently killing intracellular pathogens. However, *Leishmania* can evade these mechanisms due to expression of virulence factors. Different strains of the same *Leishmania* species may have different infectivity and metastatic phenotypes *in vivo*, and *w*e have previously shown that analysis of amastigote proteome can give important information on parasite infectivity. Differential abundance of virulence factors probably accounts for the higher virulence of PH8 strain parasites shown in this work. In order to test this hypothesis, we have quantitatively compared the proteomes of PH8 and LV79 lesion-derived amastigotes using a label-free proteomic approach.

**Methodology/Principal findings:**

In the present work, we have compared lesion development by *L*. *(L*.*) amazonensis* PH8 and LV79 strains in mice, showing that they have different virulence *in vivo*. Viability and numbers of lesion-derived amastigotes were accordingly significantly different. Proteome profiles can discriminate parasites from the two strains and several proteins were differentially expressed.

**Conclusions/Significance:**

This work shows that PH8 strain is more virulent in mice, and that lesion-derived parasites from this strain are more viable and more infective *in vitro*. Amastigote proteome comparison identified GP63 as highly expressed in PH8 strain, and Superoxide Dismutase, Tryparedoxin Peroxidase and Heat Shock Protein 70 as more abundant in LV79 strain. The expression profile of all proteins and of the differential ones precisely classified PH8 and LV79 samples, indicating that the two strains have proteins with different abundances and that proteome profiles correlate with their phenotypes.

## Author summary

Leishmaniasis is an antropozoonosis caused by *Leishmania* parasites that affects around 12 million people in 98 different countries. Cutaneous leishmaniasis caused by *Leishmania amazonensis* can have different clinical forms and severities depending on the parasite strain. We have here shown that two *Leishmania amazonensis* strains, named PH8 and LV79, which have different virulence in mice, also have different protein signatures. In fact, samples from these strains can be distinguished based on the abundance of all proteins detected and of the differential ones. Differential proteins identified in this work may be employed in the future to predict virulence of parasite strains or isolates.

## Introduction

Leishmaniasis is an antropozoonosis that affects around 12 million people in 98 different countries in Europe, Africa, Asia and America [1]. More than 1,5 million new cases are reported every year, 0,7 to 1,2 of them of the tegumentary forms and 0,2 to 0,4 million of the visceral form [1]. The clinical form of the disease depends mainly on the *Leishmania* species and on the immunologic status of the host [2]. In Brazil, *Leishmania (Viannia) braziliensis* and *Leishmania (Leishmania) amazonensis* are the species most frequently involved in tegumentary leishmaniasis [3]. The human *L*. *(L*.*) amazonensis* symptomatic infection frequently leads to the localized cutaneous leishmaniasis (LCL), with moderate cellular hypersensitivity, and more rarely to the diffuse cutaneous leishmaniasis (DCL), associated with anergy to parasite’s antigens [3].

The parasite has two main forms: promastigotes, transmitted by an infected female phlebotomine sand fly, and amastigotes, which live and replicate in phagolysosomes of phagocytic cells, mainly macrophages [4,5]. Macrophages are important cells of the immune system, capable of directly killing intracellular pathogens and triggering adaptive responses against them [6]. When activated, these cells produce cytokines and reactive oxygen species, nitric oxide, lysosomal enzymes and proteases with microbicidal effects [5]. *Leishmania*, however, can evade these mechanisms and replicate inside macrophages due to parasite´s virulence factors [7,8].

The importance of specific virulence factors may vary according to the *Leishmania* species. Protein A2, LACK (homolog of receptor for activated C kinase) and cathepsin L-like cysteine protease B (CPB), for instance, are considered important factors for *L*. *(L*.*) donovani*, *L*. *(L*.*) major* and *L*. *(L*.*) amazonensis*, respectively [2]. Inositol phosphosphingolipid phospholipase C-like (ISCL) is also considered an essential factor for *L*. *(L*.*) major* survival inside the acid phagolysosome [9]. Curiously, while *L*. *(L*.*) major* ISCL knock out parasites lost virulence in BALB/c mice, *L*. *(L*.*) amazonensis* ko parasites had similar virulence compared to wild type in this mouse strain [10].

Lipophosphoglycan (LPG) and major surface glycoprotein GP63 are by far the most studied *Leishmania* virulence factors. LPG is the most abundant molecule in promastigote´s surface [11]. It inhibits macrophage nitric oxide production, signal transduction and apoptosis, delays phagolysosome maturation and induces RNA double strand-dependent protein kinase (PKR), which increases parasite growth [12–14]. Although essential for *L*. *(L*.*) major* and *L*. *(L*.*) donovani* infectivity, LPG is not necessary for *L*. *(L*.*) mexicana* infection *in vitro* and *in vivo* [15,16]. The zinc-metalloprotease GP63 is an important antigen in promastigotes, also expressed (at lower levels) in amastigotes [17]. GP63 facilitates *Leishmania* infection and survival since it degrades extracellular matrix, decreases kinase and upregulates phosphatase activity in infected macrophages, and enhances the resistance to antimicrobial peptides. Besides, GP63 cleaves C3 to C3b and C3bi, increasing parasite resistance to complement-mediated lysis, and directly cleaves the pro-inflammatory factors AP-1 and NF-κB (reviewed in [11,17]). Interestingly, it was recently shown that cysteine peptidase B, an important virulence factor for *L*. *(L*.*) mexicana* and *L*. *(L*.*) amazonensis* [18], regulates the levels of LPG and GP63 in *L*. *(L*.*) mexicana* [19].

While some factors are restricted to the parasite surface, others can be secreted. GP63, elongation factor 1 alpha (EF-1α), frutose-1,6-bisphosphate aldolase, secreted acid phosphatase (SAcP), heat shock proteins (HSPs) 10 and 70 and tryparedoxin peroxidase, among others, are produced and secreted by amastigotes [8,20]. Not only GP63, as previously mentioned, but also EF-1α, aldolase and SAcP, interact with macrophage kinases and phosphatases, reducing cell activation and microbicidal capacity [8]. Cysteine peptidases may either accumulate inside amastigotes or be secreted in exosomes, depending on the *Leishmania* species. These important virulence factors have roles both inside the parasite and in the host [11].

It is well known that *Leishmania* species differ in terms of virulence, as illustrated by the fact that several mouse lineages are resistant to *L*. *(L*.*) major* and susceptible to *L*. *(L*.*) amazonensis* [2,21]. It is also known that strains of the same *Leishmania* species may show different infectivity and metastatic phenotypes *in vivo* [22–24]. Although proteome comparison has been extensively employed for the identification of proteins involved in resistance to drugs [25–29], few studies have used this strategy to identify virulence factors. One of them compared different clones of *L*. *(V*.*) guyanensis* and identified two proteins associated with metastatic capacity [22]. Another study analyzed two strains of *L*. *(L*.*) infantum* with different infectivity *in vivo* and found that proteins such as KMP-11, heat shock proteins, tryparedoxin peroxidase (CPx) and peroxidoxin were differentially expressed [23]. A recent work compared *L*. *(V*.*) braziliensis* isolates from mucosal and cutaneous lesions of the same patient and observed overexpression of prostaglandin f2-alpha synthase and HSP70 in cutaneous isolates [24].

We have previously shown that LV79 strain of *L*. *(L*.*) amazonensis* develop small lesions in C57BL/6 mice. In fact, LV79 lesions in this mouse strain increase until six weeks after inoculation and decrease thereafter, although parasites can still be found in lesions until thirteen weeks post infection [30]. On the other hand, PH8 strain was shown to generate lesions of increasing size in the same mouse strain [31]. In the present work, we show that promastigotes from LV79 and PH8 strains induce different lesion development in BALB/c and C57BL/6 mouse strains, and that amastigotes from PH8 are more infective. Differential abundance of virulence factors probably accounts for the higher virulence of PH8 amastigotes. In order to test this hypothesis, we have quantitatively compared the proteomes of PH8 and LV79 lesion-derived amastigotes using a label-free proteomic approach.

The comparison of the proteomes of lesion-derived amastigotes from the two strains identified proteins such as CPx, SOD and HSP70 as significantly more abundant in LV79 amastigotes, and GP63 as more abundant in PH8 parasites. The expression profile of all proteins and of the differentially expressed ones precisely classified PH8 and LV79 samples, indicating that protein abundance profiles correlate with the phenotypes of the two strains.

## Materials and methods

### Ethics statement

All animals were used according to the Brazilian College of Animal Experimentation (CONEP) guidelines, and the protocols were approved by the Institutional Animal Care and Use Committee (CEUA) of the University of São Paulo (protocol number 001/2009). Euthanasia was performed in CO2 camera.

### *Leishmania (L*.*) amazonensis* promastigotes

Promastigotes of *Leishmania (L*.*) amazonensis* LV79 (MPRO/BR/72/M 1841) and PH8 (IFLA/BR/67/PH8) strains were cultured at 24°C in M199 medium supplemented with 10% fetal calf serum (FCS). Parasites were sub-cultured every 7 days to inoculums of 2 × 10^6^/mL.

For differentiation of amastigotes into promastigotes, lesion-derived parasites were counted using Neubauer chamber and transferred to M199 medium with 10% FCS at densities of 10^3^, 10^4^ and 10^5^ parasites/mL. Cells were incubated at 24°C for 4 days and promastigote densities were determined.

### Mice infection and histopathological analysis

Four to 8-week-old BALB/c and C57BL/6 mice maintained in our facilities were infected in the left hind footpads with 2 × 10^6^ promastigotes of *L*. *(L*.*) amazonensis* LV79 or PH8 in the beginning of stationary-phase (day 5, see S1 Fig) in a final volume of 20μL. Footpad thickness was measured weekly using a caliper (Mitutoyo Corporation, Japan). For histological analysis, we employed five BALB/c animals for each parasite strain. Animals were euthanized, infected paws were removed and control footpads were removed from uninfected mice with similar ages. Fragments of these tissues were fixed in 10% buffered formalin for 18 h, washed and dehydrated in graded concentrations of ethanol, diaphanized and embedded in paraffin. The 4 μm paraffin sections were stained with hematoxylin and eosin.

For immunohistochemistry, sections were deparaffinized, blocked with 5% BSA in PBS for 30 minutes, and incubated in 0,1% sodium azide, 3% H_2_O_2_ in methanol for 30 minutes for blocking endogenous peroxidase. After incubation with rabbit anti-*Leishmania* serum (gently provided by Prof. Mauro Cortez) 1:1000 in PBS 2% (w/v) BSA for 18h at 4°C, slides were washed in PBS and incubated with secondary anti-rabbit peroxidase-conjugated antibody (Imuny, Brazil) 1:2000 in PBS 2% (w/v) BSA for two hours. Slides were then washed in PBS, incubated with DAB (DAKO, Denmark) for 2 minutes, washed in water and counterstained with hematoxylin. Samples were dehydrated and diaphanized, mounted with Permout (Sigma) and analyzed in Nikon Eclipse E200 LED microscope with Moticam 580 (Motic) camera.

### Amastigote purification and lysis

Amastigotes were purified as previously described [32]. Briefly, lesions were minced and homogenized in 5mL PBS using a tissue grinder (Thomas Scientific). After centrifugation at 50 x g for 10 min at 4°C, the supernatant was recovered and centrifuged at 1450 x g for 17 min at 4°C. Supernatant was then discarded and the pellet was washed three times with PBS followed by centrifugations at 1450 x g for 17 min at 4°C. After 3h of incubation in RPMI with 4% serum under rotation at room temperature to liberate endocytic membranes, amastigotes were further centrifuged, resuspended in 2mL of erythrocyte lysis buffer (155mM NH4Cl, 10mM KHCO3, 1mM EDTA, pH7,4) and incubated for 2 min in ice. Parasites were washed twice in PBS, resupended at 10^9^ cells/300μL in PBS + Proteoblock (a protease inhibitor cocktail from Fermentas) and lysed by 8 cycles of freeze thaw in liquid nitrogen-42°C. Soluble proteins were obtained after centrifugation at 12.000 x g for 3 min and quantified by Bradford (Biorad).

### MTT assay and trypsin-like activity

MTT assay was performed using MTT (3-[4,5-dimethylthiazol-2-yl]-2,5- diphenyltetrazolium bromide (Sigma) as previously described [13]. Briefly, 2x10^7^ lesion-derived amastigotes were resuspended in 100 μL PBS with 5mM glucose, transferred to 96 well plates and incubated with 20 μL of MTT (5mg/ml in PBS) at 34°C for 50 minutes. 100 μl of SDS 10% were added and absorbance at 595 nm (reference at 655nm) was measured in BioTek ELx800 equipment (Biotek, Winooski,VT, USA).

Trypsin-like activity in amastigote extracts was assayed as we recently described [33].

### Mass spectrometry analysis

100 μg of soluble amastigote proteins from each sample were digested with trypsin. The resulting peptide mixture was analyzed on a LTQ Velos Orbitrap mass spectrometer (Thermo Fisher Scientific) coupled with LC-MS/MS by an EASY-nLC system (Thermo Fisher Scientific) through a nanoelectrospray ion source. Sample concentration and desalting were performed online using a pre-column (2 cm; 100 μm ID; 5 μm C18-A1; Thermo). Separation was accomplished on Acclaim PepMap 100 C18 column (10cm; 75um ID; 3um C18-A2; Thermo) using a linear gradient of A and B buffers (buffer A: A = 0.1% formic acid; Buffer B = 99% ACN, 0.1% formic acid) from 1% to 50% buffer B over 60 for a total of 77 min at a flow rate of 0.3 μL/min to elute peptides into the mass spectrometer. Columns were washed and re-equilibrated between LC—MS/MS experiments. Mass spectra were acquired in the positive-ion mode over the range *m*/*z* 400–1500 at a resolution of 30,000 (full width at half-maximum at *m*/*z*400) and AGC target >1 × *e*^6^. The 20 most intense peptide ions with charge states ≥2 were sequentially isolated to a target value of 5,000 and isolation width of 2 and fragmented in the linear ion trap using low-energy CID (normalized collision energy of 35%) with activation time of 10 ms. Dynamic exclusion was enabled with an exclusion size list of 500, exclusion duration of 30 s, and a repeat count of 1. Three biological replicates (amastigotes from three independent mice infections) were performed with two technical runs for LV79 and PH8.

### Protein identification and bioinformatic analyses

For protein identification and quantification, raw files were imported into MaxQuant version 1.5.2.8 [34]. The database search engine Andromeda [34,35] was used to search MS/MS spectra against a database composed of Uniprot *Mus musculus* (release May 5^th^, 2016; 50,189 entries) and *Leishmania sp* (release May 5^th^ 2016, 50,820 entries) databases. Database search employed the following parameters: (i) mass tolerance of 4.5 ppm and 0.5 Da for MS and MS/MS, respectively; (ii) trypsin cleavage at both ends and two missed cleavage allowed; (iii) carbamidomethylation of cysteine (57.021 Da) was set as a fixed modification, and oxidation of methionine (15.994 Da) and protein N-terminal acetylation (42.010 Da) were selected as variable modifications. All identifications were filtered to achieve a protein and peptide FDR of 1%. One peptide was set as the minimum number for protein identification, and all proteins identified with one peptide had this peptide as unique peptide that could unambiguously identify that protein. For protein quantification, a minimum of two ratio counts were required. All identifications were filtered to achieve a protein and peptide FDR of less than 1% as recommended in the proteomic community for large scale mass spectrometry-based experiments acquired in the data-dependent mode used in this study.

Protein quantification was based on the MaxQuant label-free algorithm using both unique and razor peptides for protein quantification, and at least 2 ratio counts were required for considering a protein quantification valid. Protein abundance was calculated based on label-free protein quantification (LFQ) values, which are normalized intensities calculated by the MaxQuant software [36]. LFQ-based quantification was shown to provide very accurate and robust quantification and has been validated in many diverse biological contexts [37]. Fold changes were calculated by dividing the average of the LFQ intensities from LV by the average of LFQ intensities from PH replicates.

Statistical analyses of the proteome data were performed using Perseus v.1.5.4.1 in the MaxQuant environment. First, proteins identified in the reverse database, potential contaminants and proteins identified only by site were excluded. The LFQ intensities were log2 transformed and the averages of the two technical replicates values for each independent experiment were calculated. T-test analysis was applied on the PV and PH groups with a p value set to p<0.05. Hierarchical clustering of significantly altered proteins was performed using the Z-score calculation on the log2 intensity values, and the results were represented as a heat map. Principal component analysis was constructed in the web-based chemometrics platform MetaboAnalyst 2.0 [38].

### Western blot

Western blots were performed as previously described [32] using 25μg of soluble amastigote proteins and 12% acrylamide gels. After incubation with ECL Prime Western Blotting Detection Reagent (GE healthcare) for five minutes, membranes were developed using ChemiDoc XRS+ (BioRad) and analyzed using Image Lab (BioRad) software. The results were normalized to actin band intensities.

## Results

**Fig 1:**
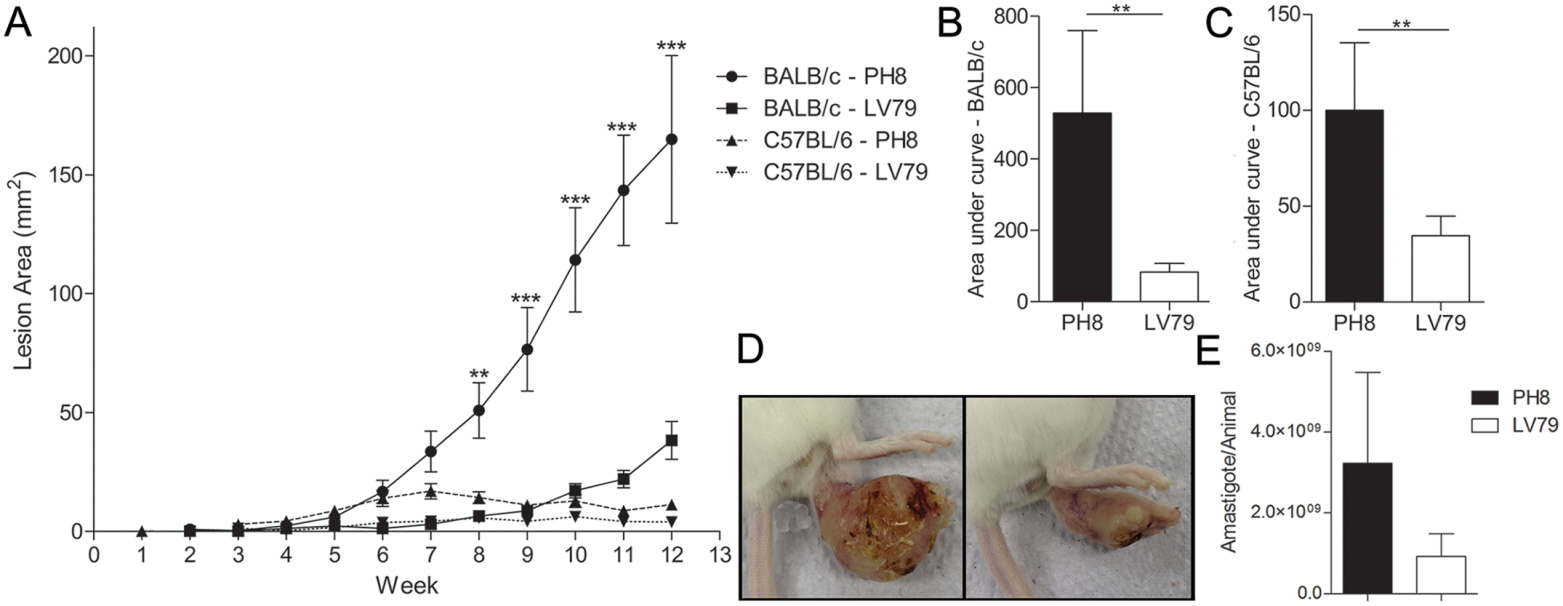
Lesions in BALB/c and C57BL/6 mice infected with *L*. *(L*.*) amazonensis* promastigotes from LV79 and PH8 strains. A. Lesion areas of BALB/c and C57BL/6 measured weekly during 12 weeks. T-test between PH8 and LV79 in the same mouse strain, **: p<0.01, ***: p<0.001. B. Area under curve for BALB/c mice infected with LV79 and PH8. C. Area under curve for C57BL/6 mice infected with LV79 and PH8, T-test, **: p<0.01. D. Images of BALB/c lesion 12 weeks p.i. with PH8 (upper) and LV79 (lower) promastigotes. E. Parasite recovery from PH8 and LV79 lesions from three independent infections in BALB/c mice. T-test.

Both LV79 and PH8 *L*. *(L*.*) amazonensis* strains cause lesions in BALB/c and C57BL/6 mice, but lesions were smaller and decreased with time in C57BL/6 mice (Fig 1A). On the other hand, BALB/c lesions were significantly larger than C57BL/6 for both parasite strains, as we have already described [30]. PH8 lesions were significantly larger than LV79 in both mouse strains (Fig 1B, 1C and 1D), and parasite loads tend to be higher in infections with this *L*. *(L*.*) amazonensis* strain (Fig 1E).

**Fig 2:**
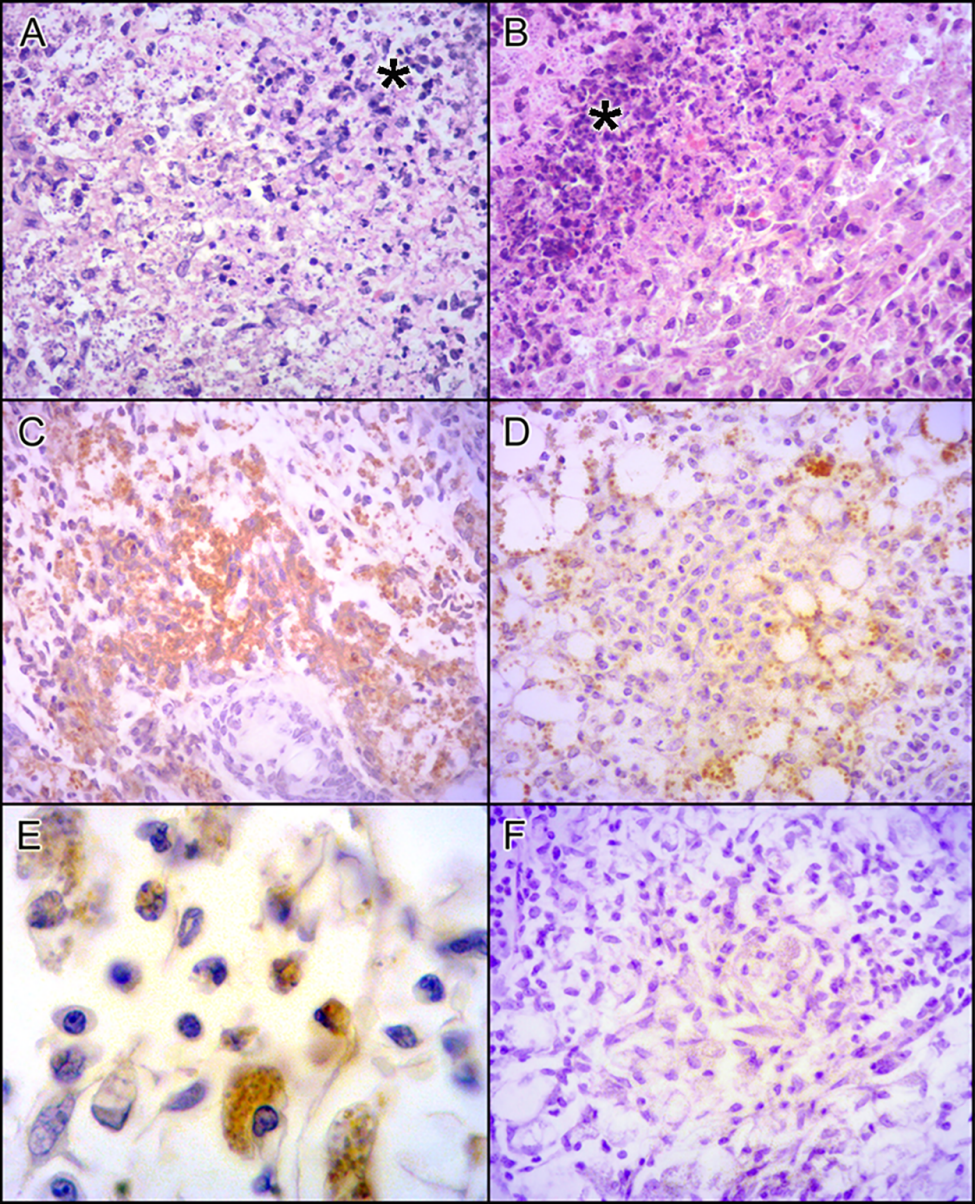
Lesions in BALB/c mice infected with *L*. *(L*.*) amazonensis* promastigotes from LV79 and PH8 strains for 12 weeks. HE staining of BALB/c mice footpads infected with PH8 (A) and LV79 (B) with necrosis area (*). Parasite labeling in PH8 (C) and LV79 (D) lesions by IHQ using anti-*Leishmania* serum and anti-rabbit HRP antibody. In E, higher magnification of IHQ of PH8 lesion, in F, negative control with secondary antibody but no anti-*Leishmania* serum. A, B, C, D and F 400X magnification, E with 1000x magnification.

We also compared histological sections of PH8 and LV79 lesions in BALB/c mice. After twelve weeks of infection, BALB/c mice showed disrupted footpad structure and high abundance of infected macrophages for both parasite strains, and more abundant necrosis in LV79 lesions (Fig 2A and 2B). Immunohistochemistry indicated higher abundance of parasites (labeled in brown) in PH8 lesions (Fig 2C versus Fig 2D), corroborating the higher parasite recovery (Fig 1E) and lesion size (Fig 1D) observed for this *L*. *(L*.*) amazonensis* strain.

**Fig 3:**
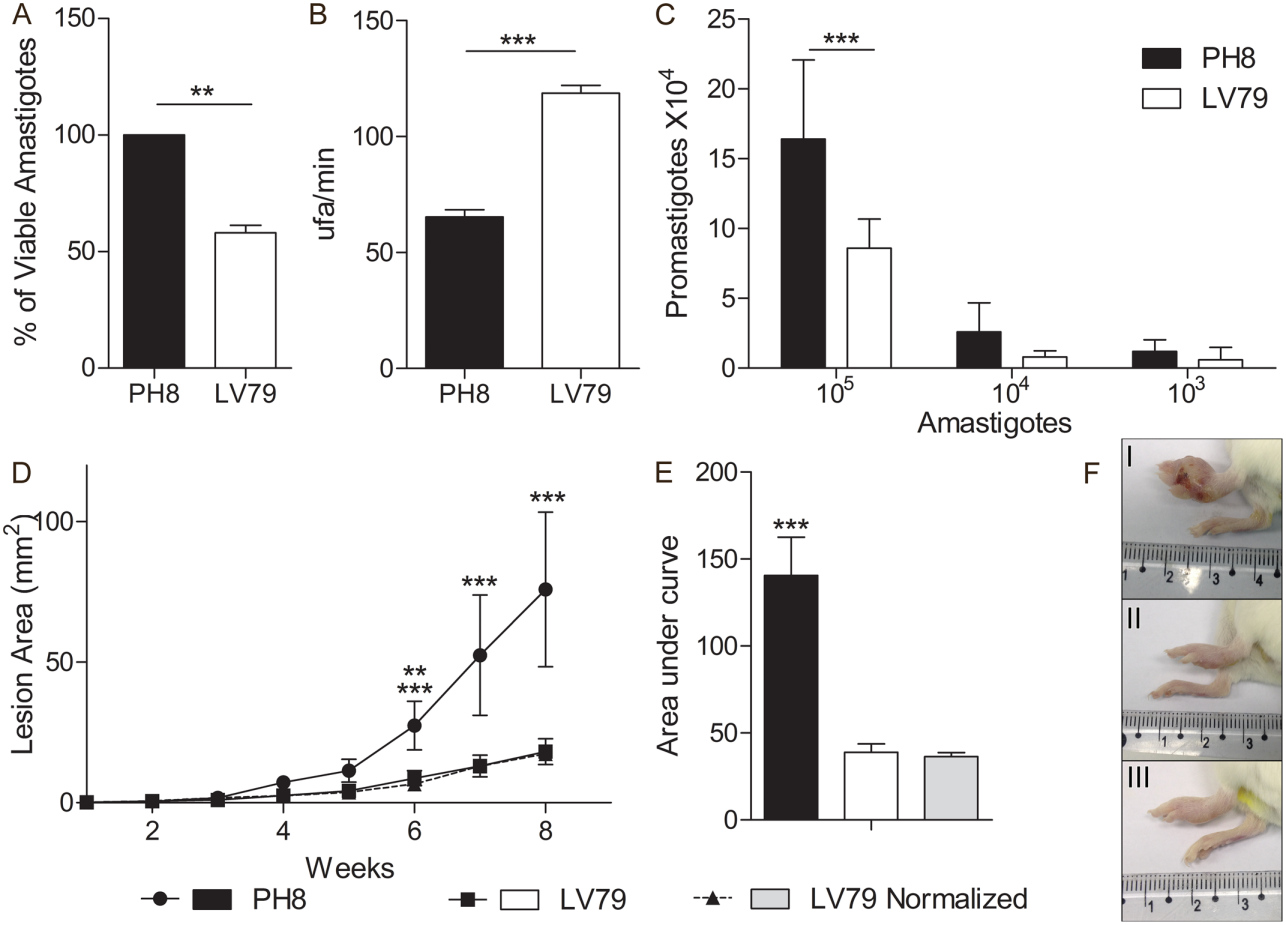
Comparison between PH8 and LV79 lesion-derived amastigotes regarding infective characteristics. A. Estimation of amastigote viability by MTT. Three independent experiments, T test, **:p<0.01. B. Trypsin-like activity of amastigote extracts. One experiment (representative of two) with technical triplicates, T test, ***:p<0.001. C. Density of promastigote cultures, indicating efficiency of conversion of amastigotes to promastigotes after 4 days in culture (one experiment with five technical replicates) T test, ***:p<0.001. D. Lesion development graph and E. the respective area under curve after infection with lesion-derived amastigotes from PH8, LV79 and normalized numbers (using viability percentages) of LV79- named as LV79 normalized. Results from three independent experiments, ANOVA followed by Tukey post test, **:p<0.01, ***:p<0.001 (6 weeks: ** for PH8 x LV79, *** for PH8 x LV79norm, 7 and 8 weeks: *** for PH8 x LV79 and LV79norm). F. Representative image in the last day of infection of BALB/c: I, infected with PH8 amastigotes, II, infected with LV79 amastigotes and III, infected with normalized numbers of LV79 amastigotes.

Infections shown in the previous experiments were initiated with promastigote cultures in stationary phase. To verify whether infections using amastigotes of PH8 and LV79 also generated lesions with significant different sizes, we isolated lesion-derived parasites and inoculated them in naïve BALB/c footpads. Before inoculation, we estimated parasite viability by MTT assay and analyzed trypsin-like activity, used as a measure of metacaspase activity, which is directly associated to parasite death [33]. We also compared parasite differentiation into promastigotes. In Fig 3A we show that lesion-derived amastigotes from PH8 strain have higher viability than LV79, and, accordingly, lower trypsin-like activity (Fig 3B). As expected, PH8 amastigotes generate cultures with higher numbers of promastigotes (Fig 3C). Lesions generated after inoculation of PH8 amastigotes were bigger than the ones generated by LV79 amastigotes, as shown in Fig 3D, 3E and 3F. To analyze if the larger sizes of PH8 lesions could be attributed to a higher number of viable parasites, we adjusted LV79 parasite numbers considering their viability, so that we would inoculate the same number of viable amastigotes for LV79 and PH8. As shown in Fig 3D, 3E and 3F, infections with normalized LV79 parasites still led to smaller lesions than PH8, indicating that the higher virulence of PH8 cannot be solely attributed to the increased viability of lesion amastigotes. In fact, only in infections using 5 or 10 times more LV79 amastigotes we observed a lesion development pattern similar to PH8´s (S2 Fig).

Differential abundance of virulence factors probably accounts for the higher virulence of PH8 amastigotes. In order to test this hypothesis, we have quantitatively compared the proteomes of PH8 and LV79 lesion-derived amastigotes using a label-free proteomic approach. Amastigote loads for LV79 strain in C57BL/6 mice lesions 13 weeks after infection are around 7 x 10^4^ parasites/footpad, much lower than the 1.5 x 10^8^ parasites/footpad of BALB/c, as we have recently shown [30]. This low parasite recovery precluded the use of C57BL/6-derived amastigotes for proteome analysis.

**Fig 4:**
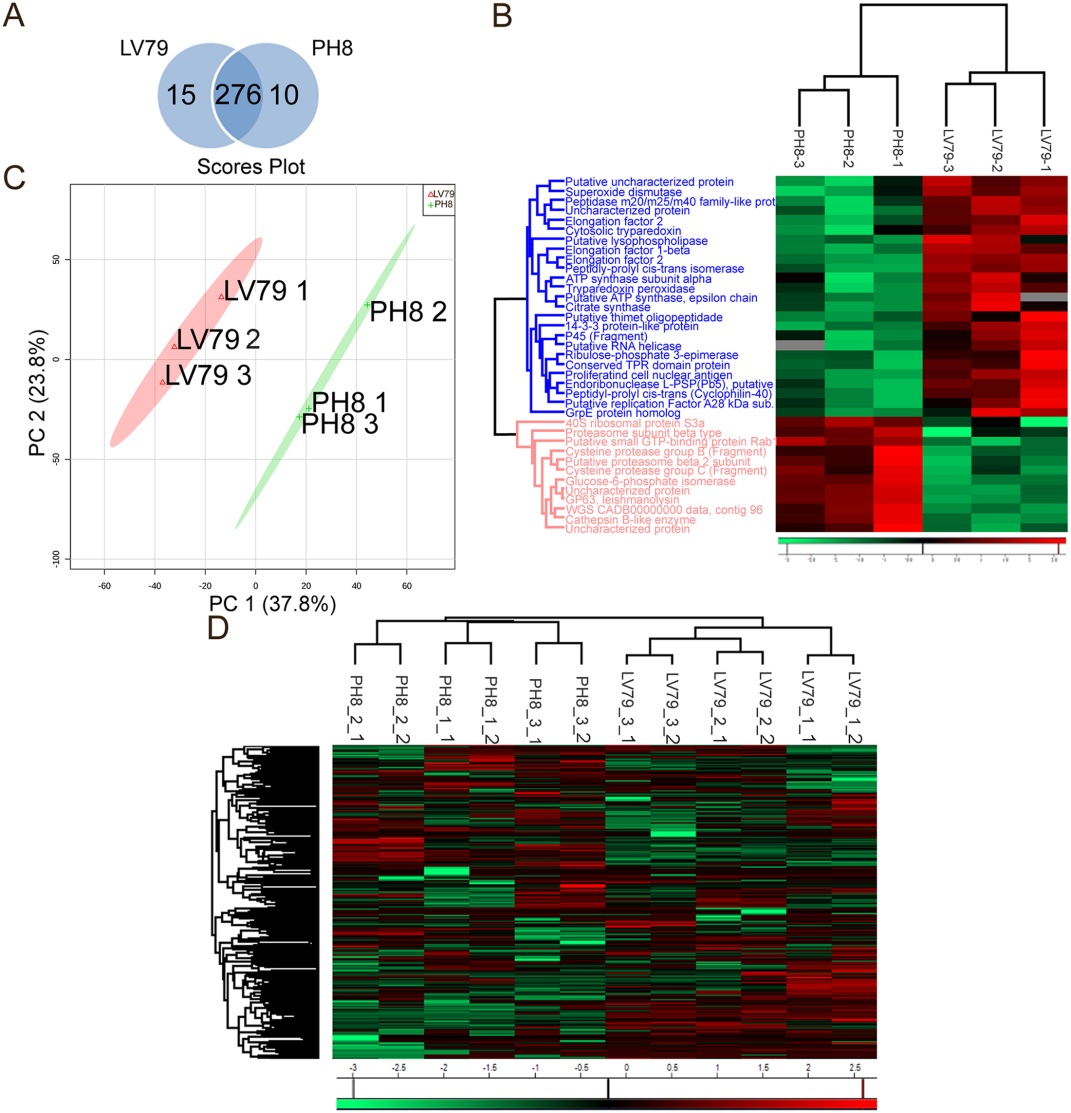
Protein comparison in PH8 and LV79 lesion-derived amastigotes. A. Venn diagram showing the overlap between all identified proteins in PH8 and LV79. B. Heat map representing log2 fold changes of the quantitative data (green—lowest abundance and red—highest abundance) of the differentially expressed proteins (T test, p <0.05). C. Main component analysis based on all proteins identified in LV79 and/or PH8. D. Heat map representing log2 fold changes of the quantitative data of all proteins identified. The first number (1 or 2) after strain name (1, 2 or 3) indicates infection experiment, the second corresponds to the technical replicate.

Three independent experiments (named 1, 2 and 3) were performed with BALB/c mice infected with stationary promastigotes of the two strains, and each amastigote sample was analyzed in technical duplicates. The total number of proteins identified in the *Leishmania* database, considering all experiments and replicates, was 301. Fig 4A indicates that 276 of the 301 proteins were detected in the proteomes of both strains, while 15 and 10 proteins were detected only in LV79 and PH8 amastigotes, respectively (S1 Table). Among the proteins identified in both samples, 12 were significantly more abundant in PH8 amastigotes and 25 in LV79 (Table 1). Among these 37 proteins, 16 had fold changes of at least 2 (ratios LV79/PH8 higher than 2 or lower than 0,5): 11 more abundant in LV79 and 5 more abundant in PH8, which are now depicted in bold in Table 1. Although most fold changes were not very high, they are robust since they have statistical significance after t-test of three independent experiments. These results indicate that among the 301 proteins identified, 20% (62 proteins) were either exclusively detected or increased in one of the strains. It is important to mention that among the 301 proteins, 218 (72%) were common across all experiments (PH8 and LV79) and replicates. We also observed that the R2 correlation value of the quantified protein signals between individual replicates was excellent, with a range of 0.929–0.975, indicating high reproducibility among replicates.

**Table 1:** Proteins with different abundance between LV79 and PH8. Protein names and uniport ID, fold change relative to PH8, and strain showing higher abundance (ED = exclusively detected). Proteins with fold changes of 2 or more in LV79/PH8 and in PH8/LV79 are depicted in bold font [39].

Description	Uniprot ID	Fold change LV79/PH8	Direction
**Tryparedoxin peroxidase**	E9BCF0	4,62	Up LV79
**Elongation factor 2**	Q4Q259	4,37	Up LV79
**Superoxide dismutase**	E9B2V9	4,20	Up LV79
**Peptidyl-prolyl cis-trans isomerase (Cyclophilin-40) putative**	E9BT02	3,73	Up LV79
**Elongation factor 1-beta**	E9B4Q3	3,26	Up LV79
**Peptidyl-prolylcis-transisomerase**	E9AXG9	2,53	Up LV79
**Citrate synthase**	E9ARK6	2,44	Up LV79
**Putative ATP synthase, epsilon chain**	Q4Q6S8	2,36	Up LV79
**P45 (Fragment)**	Q9U8C3	2,13	Up LV79
**Peptidase m20/m25/m40 family-like protein (Fragment)**	E9B1Y8	2,10	Up LV79
**Putative replication Factor A 28 kDa subunit**	A4H800	2,00	Up LV79
ATP synthase subunit alpha	Q4QJF1	1,99	Up LV79
Ribulose-phosphate 3-epimerase	E9B3Z3	1,95	Up LV79
Putative thimet oligopeptidase	A4HF12	1,88	Up LV79
Putative RNA helicase	A4HC04	1,88	Up LV79
Putative uncharacterized protein	E9AY45	1,87	Up LV79
Conserved TPR domain protein	E9B146	1,72	Up LV79
Putative lysophospholipase	Q4QAE7	1,61	Up LV79
Elongation factor 2	E9ASD6	1,58	Up LV79
Proliferating cell nuclear antigen	Q4QF35	1,54	Up LV79
Grp E protein homolog	E9B0J8	1,51	Up LV79
14-3-3 protein-like protein	E9AT99	1,49	Up LV79
Uncharacterized protein	E9B3H9	1,45	Up LV79
Endoribonuclease L-PSP (Pb5), putative	E9BG42	1,42	Up LV79
Cytosolic tryparedoxin	A8I4U5	1,28	Up LV79
Proteasome subunit beta type	E9B5M9	0,73	Up PH8
Putative proteasome beta 2 subunit	E9ASE9	0,70	Up PH8
Cysteine protease group B (Fragment)	Q9TWP0	0,58	Up PH8
Uncharacterized protein	E9AV52	0,57	Up PH8
40S ribosomal protein S3a	E9BRS2	0,57	Up PH8
WGS CADB00000000 data, contig 96	E8NHS3	0,55	Up PH8
Putative small GTP-binding protein Rab1	E9AYX8	0,53	Up PH8
**Cysteine protease group C (Fragment)**	Q9TWN9	0,50	Up PH8
**Glucose-6 phosphate isomerase**	E9ANY1	0,47	Up PH8
**GP63, leishmanolysin**	E9AN56	0,34	Up PH8
**Cathepsin B-like enzyme**	Q25319	0,31	Up PH8
**Uncharacterized protein**	E9AS67	0,25	Up PH8
Putative uncharacterized protein	E9AP69		ED LV79
Heat Shock Protein 70 (Fragment)	A0A075FJL9		ED LV79
Peptidyl-prolylcis-trans isomerase	A4HLM4		ED LV79
6- phosphogluconolactonase	E9AYQ1		ED LV79
Putative peroxisomal enoyl-coa hydratase	Q4QDY4		ED LV79
Putative uncharacterized protein	E9B709		ED LV79
Putative mitogen-activated protein kinase	Q4QDK6		ED LV79
Putative RNA-binding protein	E9AK98		ED LV79
ATP synthase, epsilon chain, putative	A0A088RWN4		ED LV79
Tryparedoxin	E9ADX4		ED LV79
Uncharacterized protein	E9B476		ED LV79
Uncharacterized protein	E9BFN7		ED LV79
Putative pyruvate dehydrogenase E1 beta subunit	E9AXQ3		ED LV79
Putative pyruvate phosphate dikinase	Q4QGX9		ED LV79
Putative farnesyl pyrophosphate synthase	E9AVW8		ED LV79
Malate dehydrogenase	Q4Q3J5		ED PH8
Clathrin heavy chain	Q4Q1R2		ED PH8
Na/H antiporter-likeprotein	A4HCV3		ED PH8
Putative GTP-binding protein	Q4Q9V1		ED PH8
Eukaryotic translation initiation factor 6	Q4Q1Y7		ED PH8
Uncharacterized protein	E9AZ36		ED PH8
Peptidyl-prolylcis-transisomerase	E9BT84		ED PH8
Uncharacterized protein	E9B1E2		ED PH8
Putative tryparedoxin	E9ALV8		ED PH8
Eukaryotic translation initiation factor 3 subunit 7-like	A4HIN8		ED PH8

The pattern of expression of the 37 differential (but not exclusively detected) proteins precisely clustered PH8 and LV79 samples in two separate branches, as shown in Fig 4B. When we employed expression data of all identified proteins, including the two technical replicates of each sample, PH8 and LV79 samples still clustered (Fig 4D). Samples were also efficiently grouped based on principal component analysis (Fig 4C), indicating that the two strains have remarkable differences in terms of protein abundance.

Proteins with different abundance comparing PH8 and LV79 are involved in several cellular processes, among them metabolism/ ATP synthesis, signaling, proliferation/replication, translation, and oxidative stress (Table 1). These proteins included some known *Leishmania* virulence factors such as cysteine protease, tryparedoxin and tryparedoxin peroxidase (CPx), superoxide dismutase (SOD), GP63, heat shock protein 70 (HSP70) and elongation factor.

**Fig 5:**
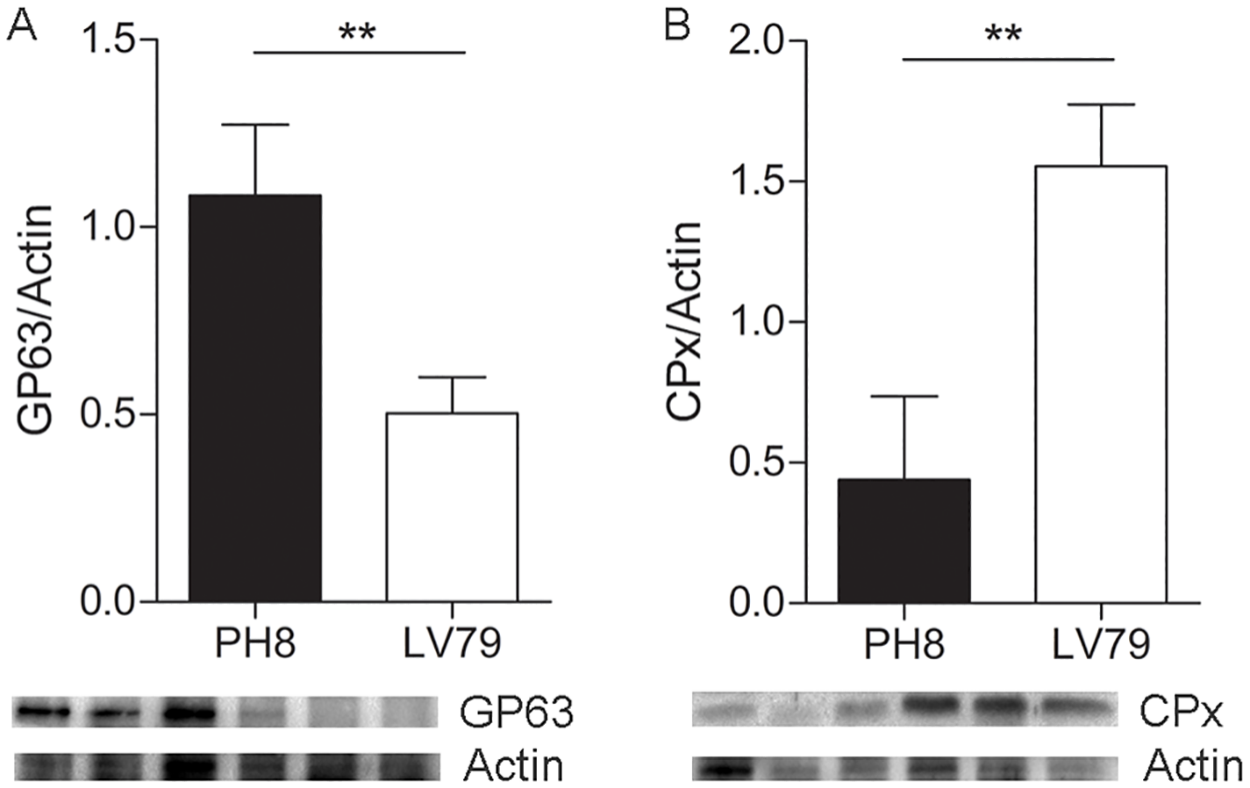
Validation of proteome data by Western blot. Western blot images and corresponding graphs showing expression of A. GP63, B. CPx in three samples of amastigotes of PH8 and LV79 strains. Statistical analysis by T test, **:p<0.01.

Proteins showing subtle differences are more difficult to validate in “semi-quantitative” Western blot assays, and for this reason we have chosen to validate proteins with ratios higher than 2: tryparedoxin peroxidase, with fold 4,62 in LV79/PH8, and GP63, with fold 0,34 in LV79/ PH8 (2,94 times more abundant in PH8). Both were analyzed using antibodies developed against *Leishmania* (anti-GP63, anti-CPx). The images and corresponding bar graphs shown in Fig 5 validate proteome analysis (Table 1): GP63 is indeed more abundant in PH8 proteomes, and CPx is more abundant in LV79 proteomes.

## Discussion

We have shown that BALB/c and C57BL/6 mice infected with promastigotes of LV79 and PH8 strains develop lesions with striking different sizes according to the parasite and mice strains. In comparison to BALB/c mice, C57BL/6 lesions were smaller and decreased with time for both parasite strains, different from the huge increasing lesions previously reported for PH8 [31]. This discrepancy may be attributed to C57BL/6 strain maintained in different animal facilities or to the parasite strain from different labs. Anyway, lesions caused by PH8 strains were significantly smaller than the ones induced by LV79 in both BALB/c and C57BL/6 mice.

Amastigotes from the two strains were compared in terms of protein abundance, as shown by proteome analysis. The 301 *Leishmania* proteins identified in this study represent a small fraction of the 6000 proteins predicted to be expressed in amastigotes, and several reasons may explain this fact. First, the study of lesion-derived amastigotes´ proteome presents some technical challenges due to the interference of host proteins, which are carried along amastigote purification and protein extraction steps even using a well stablished protocol such as ours. The presence of host proteins certainly diminishes our capacity of identifying a higher number of parasite proteins. In fact, after protein identification using a database composed of Uniprot *Mus musculus* and *Leishmania sp*, a total of 213 and 301 proteins were identified in the mouse and *Leishmania* databases, respectively, and 815 of the peptides detected belong to mouse proteins and 875 to *Leishmania* proteins. Moreover, we have analyzed the iBAQ values, which may be used as a measure of protein abundance [40], and are calculated by dividing the total intensity of a protein by the number of tryptic peptides between 6 and 30 amino acids in length. Comparing the total iBAQ value for *Leishmania* proteins to the total iBAQ value of mouse proteins, we found that *Leishmania* proteins accounted for the double of the iBAQ value of mouse ones. Besides, we did not perform sub-cellular fractionation or peptide fractionation prior to the LC-MSMS analyses. Instead, we only considered soluble proteins from a non-detergent-based protein extraction, since our main interest was on soluble amastigote virulence factors that could modulate macrophage infection and parasite survival. This strategy probably leads to a lower number of proteins compared to total extract preparations using detergents [41] or sub-cellular fractionation. At last, biological or chemical post-translational modifications as well single nucleotide polymorphism were not included as variable modifications in the MSMS search, which may represent a fraction of MSMS that was not identified.

Proteins considered as virulence factors in *Leishmania* such as CPx, SOD, GP63 and HSP70 were identified as differentially expressed between the two parasite strains. SOD, CPx and HSP70 are known to reduce oxidative damage in *Leishmania*. SODs are important in antioxidant defense in many organisms, metabolizing superoxide (O2-) into oxygen (O2) and hydrogen peroxide (H_2_O_2_). They are organized in three families based on the metal ion that supports activity: Ni, Cu complexed with Zn, and Mn or Fe [42]. Eukaryotes including mammals have Cu/ Mn/ ZnSODs, whereas FeSODs have been found in prokaryotes, protozoans, plants, and algae [43]. Different FeSOD species (FeSOD-A and FeSOD-B) have been characterized in *L*. *(L*.*) chagasi*, *L*. *(L*.*) tropica*, and *L*. *(L*.*) donovani* [44–46], and in this work we have identified a Fe SOD in *L*.*(L*.*) amazonensis* proteome similar to *L*. *(L*.*) mexicana* enzyme.

CPx has been shown to increase oxidative resistance in *L*. *(L*.*) donovani* [47], *L*. *(L*.*) infantum* [48] and *L*. *(L*.*) amazonensis* [49]. This enzyme also augments infection [47] and virulence [23] of *L*. *(L*.*) donovani*. High levels of the enzyme were reported in antimony resistant *L*. *(L*.*) donovani* [48], *L*. *(L*.*) braziliensis* and *L*.*(L*.*) chagasi* [29], in *L*. *(L*.*) amazonensis* resistant to arsenite [49] and in metastatic *L*. *(V*.*) guyanensis* [50]. HSP70 also protects *Leishmania* from toxic environmental conditions reducing heat-induced denaturation and cell death [51]. Indeed, HSP70 has been shown to be increased in *L*. *(L*.*) infantum* and *L*. *(L*.*) donovani* under heat shock or oxidative and nitrosative stresses [23,51], and the overexpression of this protein conferred increased resistance to H2O2 in *L*. *(L*.*) donovani* [51] and in *L*. *(L*.*) amazonensis* [9]. Like CPx, HSP 70 is overexpressed in antimonial resistant *L*. *(L*.*) infantum* and *L*. *(V*.*) braziliensis* parasites [29]. Besides, more virulent isolates of *L*. *(V*.*) braziliensis* showed increased HSP70 expression [24]. SOD, CPx and HSP70 were all more abundant in LV79 amastigotes. Interestingly, parasites from this strain generated smaller lesions and showed lower viability after isolation from lesions. It is possible that other virulence factors compensate for the lower expression of these three proteins and account for PH8 higher virulence and survival in the host, or that post translation modifications of one or some of these proteins generate more active protein species in PH8. In fact, we have previously described different species of CPx and HSP70 in *L*. *(L*.*) amazonensis* amastigotes [32], and HSP70 activity is known to be influenced by phosphorylation at specific residues [52].

Among the virulence factors mentioned above, only GP63 had higher abundance in the most virulent PH8 strain. Considering that this molecule favors binding of promastigotes to macrophages and intramacrophage survival and replication [53], as well as parasite survival in BALB/c mice [54], it is conceivable that a higher abundance of GP63 may contribute to PH8 virulence.

The results presented here show that amastigotes from *L*. *amazonensis* strains PH8 and LV79, which have different virulence in mice, also have proteins with different abundances. To our knowledge, this is the first gel free proteome of lesion-derived amastigotes. Despite the difficulties of working with lesion-derived parasites and the detection of a relatively low proportion of the predicted products, the comparison of PH8 and LV79 strains enabled the reproducible identification of several proteins that distinguish the two strains and that may be involved in virulence in *L*. *amazonensis*. In fact, samples from the same strain are efficiently grouped using expression data from all proteins and from the differentially expressed ones. These results indicate that PH8 and LV79 can be distinguished by comparison of protein abundances and that proteome analysis may be used to characterize *Leishmania* phenotype and eventually predict the virulence of other *L*. *(L*.*) amazonensis* strains or isolates.

## Supporting information

S1 Fig Growth curves of *L*. *amazonensis* PH8 and LV79 promastigotes in 199 medium. (TIF)

S2 Fig Lesions in BALB/c mice infected with *L*. *(L*.*) amazonensis* lesion-derived amastigotes from LV79 and PH8 strains. A. Lesion areas measured weekly during 9 weeks for infections with PH8, LV79, LV79 normalized, LV79 5x and LV79 10x amastigotes. B. Area under curve for each condition mentioned in (A). Statistical analysis by ANOVA followed by Tukey post test, *:p<0.05, **:p<0.01, ***:p<0.001 (7 weeks: * for LV79 x PH8 and LV79 5x, ** for LV79 10x versus LV79 and LV79norm, 8 weeks: *** for PH8 x LV79 and LV79norm, LV79 x LV79 5x and LV79 10x, LV79norm x LV79 5x and 10x, 9 weeks: *** for PH8 x LV79 and LV79norm, LV79 x LV79 5x and LV79 10x, LV79norm x LV79 5x and LV79 10x).
(TIF)

S3 Fig Membrane staining with Ponceau. (TIF)

S1 Table Protein groups identified in the soluble proteome of PH8 and LV79 amastigotes. (XLSX)

S2 Table Protein groups identified in the soluble proteome of PH8 and LV79 amastigotes after reverse and potential contaminants were excluded as well as proteins identified in mouse database. The LFQ intensities was log2 transformed and T-test was applied in PH8 and LV79 group samples (* = p<0.05).
(XLSX)
