# Supplementary figures and images for: Quantitative proteomic analysis of amastigotes from Leishmania (L.) amazonensis LV79 and PH8 strains reveals molecular traits associated with the virulence phenotype
Source: PLoS Negl Trop Dis. 2017 Nov 27;11(11):e0006090. doi: 10.1371/journal.pntd.0006090 (PMC5720813; doi:10.1371/journal.pntd.0006090)

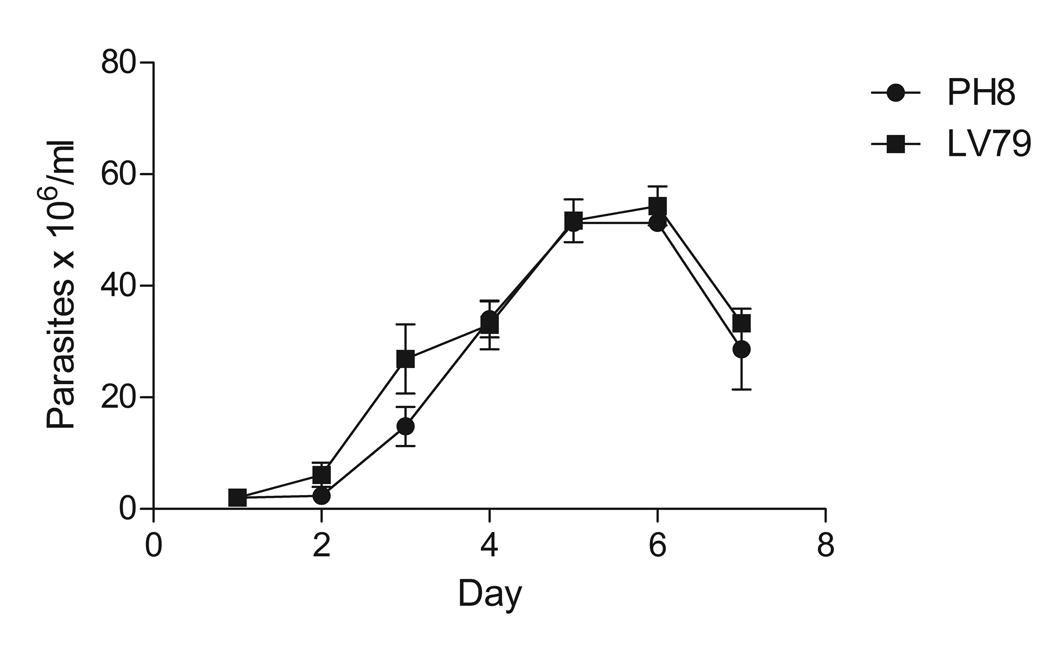

Supplement: S1 Fig — (TIF) [file pntd.0006090.s001.tif]

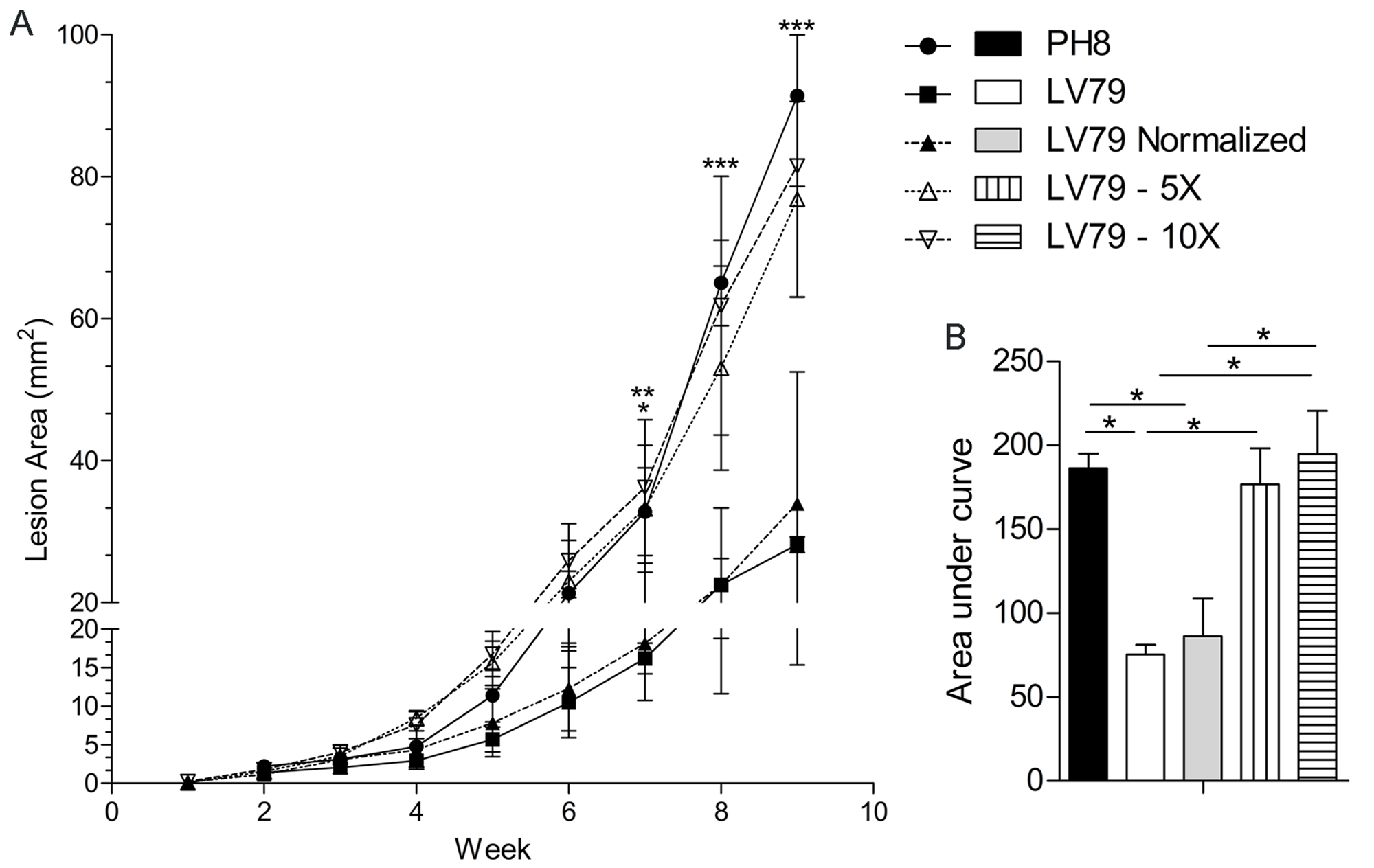

Supplement: S2 Fig — A. Lesion areas measured weekly during 9 weeks for infections with PH8, LV79, LV79 normalized, LV79 5x and LV79 10x amastigotes. B. Area under curve for each condition mentioned in (A). Statistical analysis by ANOVA followed by Tukey post test, *:p<0.05, **:p<0.01, ***:p<0.001 (7 weeks: * for LV79 x PH8 and LV79 5x, ** for LV79 10x versus LV79 and LV79norm, 8 weeks: *** for PH8 x LV79 and LV79norm, LV79 x LV79 5x and LV79 10x, LV79norm x LV79 5x and 10x, 9 weeks: *** for PH8 x LV79 and LV79norm, LV79 x LV79 5x and LV79 10x, LV79norm x LV79 5x and LV79 10x). (TIF) [file pntd.0006090.s002.tif]

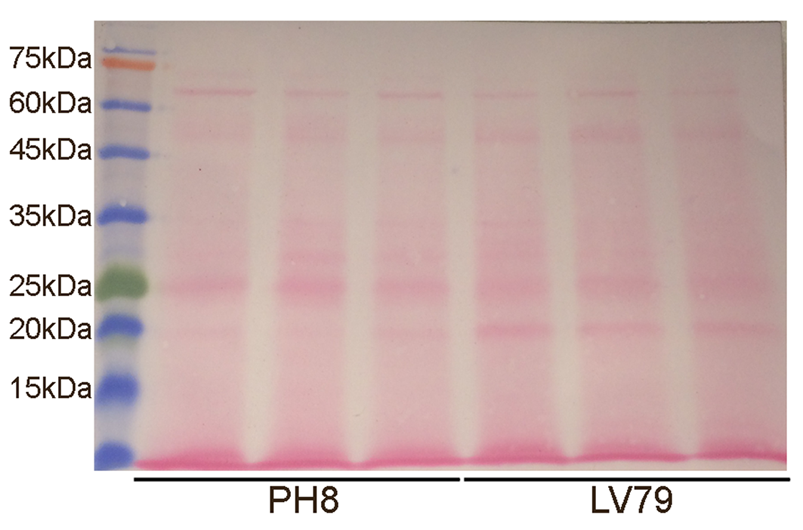

Supplement: S3 Fig — (TIF) [file pntd.0006090.s003.tif]
